# Predicting Cardiovascular Risk Using Social Media Data: Performance Evaluation of Machine-Learning Models

**DOI:** 10.2196/24473

**Published:** 2021-02-19

**Authors:** Anietie U Andy, Sharath C Guntuku, Srinath Adusumalli, David A Asch, Peter W Groeneveld, Lyle H Ungar, Raina M Merchant

**Affiliations:** 1 Penn Medicine Center for Digital Health University of Pennsylvania Philadelphia, PA United States; 2 Leonard Davis Institute of Health Economics University of Pennsylvania Philadelphia, PA United States; 3 Penn Medicine Center for Health Care Innovation University of Pennsylvania Philadelphia, PA United States; 4 Division of Cardiovascular Medicine Perelman School of Medicine University of Pennsylvania Philadelphia, PA United States; 5 Center for Health Equity Research and Promotion Corporal Michael J Crescenz VA Medical Center Philadelphia, PA United States; 6 Department of Medicine Perelman School of Medicine University of Pennsylvania Philadelphia, PA United States; 7 Department of Emergency Medicine Perelman School of Medicine University of Pennsylvania Philadelphia, PA United States

**Keywords:** ASCVD, machine learning, natural language processing, atherosclerotic, cardiovascular disease, social media language, social media

## Abstract

**Background:**

Current atherosclerotic cardiovascular disease (ASCVD) predictive models have limitations; thus, efforts are underway to improve the discriminatory power of ASCVD models.

**Objective:**

We sought to evaluate the discriminatory power of social media posts to predict the 10-year risk for ASCVD as compared to that of pooled cohort risk equations (PCEs).

**Methods:**

We consented patients receiving care in an urban academic emergency department to share access to their Facebook posts and electronic medical records (EMRs). We retrieved Facebook status updates up to 5 years prior to study enrollment for all consenting patients. We identified patients (N=181) without a prior history of coronary heart disease, an ASCVD score in their EMR, and more than 200 words in their Facebook posts. Using Facebook posts from these patients, we applied a machine-learning model to predict 10-year ASCVD risk scores. Using a machine-learning model and a psycholinguistic dictionary, Linguistic Inquiry and Word Count, we evaluated if language from posts alone could predict differences in risk scores and the association of certain words with risk categories, respectively.

**Results:**

The machine-learning model predicted the 10-year ASCVD risk scores for the categories <5%, 5%-7.4%, 7.5%-9.9%, and ≥10% with area under the curve (AUC) values of 0.78, 0.57, 0.72, and 0.61, respectively. The machine-learning model distinguished between low risk (<10%) and high risk (>10%) with an AUC of 0.69. Additionally, the machine-learning model predicted the ASCVD risk score with Pearson *r*=0.26. Using Linguistic Inquiry and Word Count, patients with higher ASCVD scores were more likely to use words associated with sadness (*r*=0.32).

**Conclusions:**

Language used on social media can provide insights about an individual’s ASCVD risk and inform approaches to risk modification.

## Introduction

Secondary prevention approaches have improved the longevity of patients with cardiovascular (CV) disease; however, risk factors and adverse health behaviors (eg, physical inactivity, smoking) are highly prevalent, and <1% of adults in most contemporary series meet all factors of ideal CV health [1]. The logistics and practicalities of meeting the goal of ideal CV health have not been clearly elucidated. Practice guidelines recommend using atherosclerotic cardiovascular disease (ASCVD) pooled cohort equations (PCEs) [2] or other prediction tools to classify patients’ risk of CV disease and the need for risk-reducing therapies such as statin medications [3]. There is also an increasing focus on identifying markers that provide better measures of risk. To best prevent ASCVD, it is important to precisely determine an individual’s 10-year risk for ASCVD. As digital platforms are increasingly used to document health behaviors, data from digital sources may provide a window into manifestations of novel risk factors and can provide complementary data to characterize existing risk factors.

Social media data in the form of posts, photos, and “likes” can provide information about individuals’ daily activities and behaviors. Social media has been used to track heart disease mortality rates [4] and depression [5]. Data on social media platforms are generated at a fast rate. Accessing these data from consenting individuals offers an opportunity to collect and analyze these data in real time. This information could facilitate identification of earlier signals of disease development or exacerbation, and timely tracking of the health of individuals and the collective health of a community [4-9]. The data are generally unscripted and spontaneous, and can therefore provide information that is different from standard survey assessments. Another potential use of data from digital platforms is that they can be used for direct intervention, so that the same platforms that are being used to assess insights can also be used to deliver targeted health information or evaluate information delivery.

The potential of social media data for CV health lies in tracking, codifying, and better understanding the hard-to-measure lifestyle choices, along with exposures related to diet, exercise, smoking, and other factors that can significantly contribute to the development and progression of heart disease. At present, measuring many of these behaviors is dependent on self-report and recall [4]. Yet, posts or images from digital media could better inform a patient-provider discussion about how to change actual dietary choices and consumption. Incorporating data from digital sources has the potential to enhance our approach for characterizing individuals’ risk and tailoring management, as a new type of precision medicine.

We sought to use social media data from consenting individuals to predict ASCVD risk reported in an electronic medical record (EMR), and to characterize differences in posts relative to four categories based on the 10-year primary risk from ASCVD risk scores.

## Methods

### Data and Design

This was a retrospective analysis of social media and EMR data of consenting patients. This study was approved by the University of Pennsylvania Institutional Review Board.

Recruitment for compiling the social mediome dataset began in March 2014 and included patients from inpatient and outpatient settings across two urban academic medical centers. Participants in this dataset consented to sharing access for selecting historical data from their social media accounts (eg, Facebook, Twitter, Instagram) and access to their medical record data. Data are stored in a Health Insurance Portability and Accountability Act (HIPAA)-compliant secure server and participants can elect to discontinue sharing data at any time point. Additional information about this dataset is published elsewhere [10,11].

Following recommendations of Goff et al [12], which outlines the process of developing a risk equation for predicting the 10-year ASCVD risk of individuals between the ages of 40 and 79 years, from our dataset, we identified patients aged 40 to 79 years and without a prior history of ASCVD documented in their EMR. Of these patients, we identified 181 with a calculated 10-year primary risk of ASCVD score in their EMR. For all these patients, demographics (age, race, gender) were also extracted from the EMR along with ASCVD scores. We retrieved Facebook status updates up to 5 years prior to enrollment for all users.

Table 1 shows the demographic information of patients included in our analysis. Of the 181 participants meeting the criteria for this study, the majority were women and Black, and the average age was 50 years. The participants had 159,958 Facebook posts overall (mean 884, SD 3227).

**Table 1:** Demographic information of patients included in our analysis (N=181).

Characteristic			Value
**Race, n (%)**			
	African American	104 (57.5)	
	White	64 (35.4)	
	Other	13 (7.2)	
Men, n (%)		48 (26.5)	

We used two approaches to process language from social media posts for inclusion in a regression model. Specifically, language features from posts were derived using (a) open vocabulary topics and (b) dictionary-based psycholinguistic features. These derived language features were then used to predict the patients’ 10-year ASCVD risk scores and to distinguish patients with different ASCVD risk scores.

### Open Vocabulary Approach

The open vocabulary approach uses latent Dirichlet allocation (LDA) [13], which is a natural language-processing method that is used to analyze the co-occurrences of words in text (in this case Facebook posts). Distinct groupings of these words represent topics (eg, groups of co-occurring words) and these topics can be labeled based on their content. For example, the model could cluster the words “dinner,” “cheese,” “eat,” “made,” and “food” as a reference to food by utilizing the similarities in the distributional properties in the Facebook posts. We generated 20 topics using Facebook posts from all of the users in our dataset. Each user was represented as a 20-dimensional vector based on the probability of each topic in all users’ posts. Multimedia Appendix 1 shows the LDA topics and 10 words associated with each topic. To determine the number of topics, consistent with prior work [14,15], we varied the number of topics using 10, 20, 50, 75, and 100 topics, respectively; 20 topics had the most coherent topic themes when reviewed by one of the coauthors.

### Dictionary-Based Approach

The dictionary-based psycholinguistic approach uses language from Facebook posts to identify the prevalence of predefined word categories represented in the Linguistic Inquiry and Word Count (LIWC) dictionary [16]. LIWC represents a dictionary of 73 different psycholinguistic word categories such as topical categories and emotions. For each user, the rate of words that occurred in a given LIWC category was measured and included as input in a model to predict ASCVD risk as described below.

### Predicting ASCVD Risk Scores Using Social Media Language

We sought to investigate the discriminatory power of predicting a patient’s 10-year ASCVD risk using language features derived from Facebook posts. We extracted the features described using an open-vocabulary approach and trained a logistic regression model, as implemented in Python 3.4 scikit-learn [17], to predict ASCVD risk scores using 5-fold cross-validation. We defined the outcome in three different ways.

In 2013, the American Heart Association and American College of Cardiology put forth the ASCVD PCEs [2], which can be used to predict an individual’s 10-year risk of ASCVD. Therefore, in Model 1, ASCVD risk was set as a categorical variable. We categorized patients into the following different thresholds: <5%, 5%-7.4%, 7.5%-9.9%, and ≥10%. We trained a multiclass logistic predictive model to predict these four categories of ASCVD risk scores. The prediction performance is reported as the area under the receiver operating characteristic curve (AUC).

For Model 2, the ASCVD risk score of patients was applied as a continuous variable rather than as a categorical variable that was used in Model 1. The performance of Model 2 was assessed using the Pearson correlation coefficient (*r*).

Identifying patients with high risk (≥10%) of ASCVD is of interest to clinicians. Therefore, in Model 3, we treated ASCVD risk as a dichotomous variable and built a logistic regression model to distinguish the high-risk category using language compared to low ASCVD scores (ie, <10%). Additionally, we used LIWC to distinguish the different features associated with high-risk patients by correlating the LIWC category feature of patients from their social media posts and whether they are in the high-risk (>10%) or low-risk (<10%) categories; we measured the effect size using Cohen *d*. To indicate significant correlations, we used Benjamini-Hochberg *P* value correction with a significance threshold of *P*<.001.

## Results

### Predicting ASCVD Risk Score Using Social Media Language

#### Model 1

The multiclass logistic regression model on Facebook posts was trained to classify patients in four different categories (<5%, 5%-7.4%, 7.5%-9.9%, ≥10%) based on their ASCVD risk scores. The model was able to delineate patients in the lowest risk category (<5%) from patients in other categories with an AUC of 0.78. The model delineated patients in the categories 5%-7.4%, 7.5%-9.9%, and ≥10% from those in other categories, as shown in Table 2.

**Table 2:** Area under the curve (AUC) scores for each category of atherosclerotic cardiovascular disease risk scores from Model 1.

Category	AUC (age only)	AUC (language only)
<5%	0.52	0.78
5%-7.4%	0.55	0.57
7.5%-9.9%	0.45	0.72
≥10%	0.59	0.61

#### Model 2

Using the linear regression model on Facebook posts, we predicted the ASCVD risk score of patients with *r*=0.26 (*P*<.001).

#### Model 3

The logistic regression model delineated patients with a high risk (≥10%) of ASCVD from those with a low risk (<10%) with an AUC of 0.69.

### Identifying Differentially Expressed Language Features According to High and Low ASCVD Scores

The sadness LIWC category was most strongly associated with the high ASCVD risk category (≥10%) at a Benjamini-Hochberg–corrected significance level of *P*<.001 and Pearson *r*=0.32. None of the other LDA topics or LIWC categories was significantly associated with high and low ASCVD risk.

## Discussion

### Principal Findings

Language from Facebook posts has the potential to distinguish patients based on their calculated 10-year ASCVD risk score categorization and actual risk score. Although social media data are unlikely to replace traditional approaches for predicting CV risk, these findings suggest that such data can potentially provide supplemental information about an individual’s lifestyle and behavior, which can complement our understanding of contributors to long-term CV risk. More than 2 billion people share information about their daily lives on social media platforms, which can include information about what they eat and drink, if they smoke, when they exercise, what their lab results are, and other factors associated with Life’s Simple 7 [18]. However, less is known about how much of this information is noise or if there is an actual relevant signal in the volumes of data in online chatter such as Facebook, where individuals often reveal information about themselves. Additionally, prior work has demonstrated that social media data can be used to predict several medical conditions such as diabetes and mental health conditions [4,5].

The potential opportunity in exploring social media data is that this emerging data source could include data about behavior and lifestyle that might not have been reported to clinicians. There is still a gap in how this would be implemented in clinical practice, and would require further evaluation of feasibility, acceptability, and interpretability. These data are unlikely to replace the existing risk score input but rather may provide complementary adjunct data. Prior work has explored the contribution of nonclinical factors (eg, patient interviews about socioeconomic status, health status, adherence, psychosocial characteristics) in predicting CV outcomes (eg, congestive heart failure readmissions). The model performance overall was poor, although patient-reported information extended the predicted ranges of rates of readmission and slightly improved model discrimination [19]. Social media data in the form of photos, videos, and likes [20,21] have been used to predict users’ personality [22], mental health, and other behaviors. Consequently, future work could use multiple modalities of user-generated content to model the ASCVD risk score.

In our patient cohort, a high ASCVD risk score was associated with increased use of “sad” language on Facebook. This is consistent with research demonstrating that depression is more prevalent in populations with CV disease, and is predictive of adverse outcomes (such as myocardial infarction and death) among populations with preexisting CV disease [23].

In our analysis, the AUC for Model 1 indicated low accuracy. A potential reason for this is that we used data from individuals between the ages of 40 and 79 years, and individuals in this age group do not post as much on social media compared to younger individuals. Accordingly, in our dataset, some users had fewer posts, leading to low accuracy from the AUC. We hypothesize that with more posts (ie, more words), our models will perform better.

We compared Models 1 and 3 together to determine which performed better at predicting the ASCVD risk score of individuals. Toward this end, we computed the micro AUC of Model 1 and compared it to that of Model 3, which was 0.66 and 0.69, respectively. This suggests that Model 3 is more reliable at predicting ASCVD risk compared to Model 1.

The findings of this study offer promise for using emerging digital data sources for identifying risk factors. This moves beyond what is simply reported by patients to what may be revealed when looking at a diary of information over multiple time points. This could aid clinicians in providing individualized recommendations for managing risk factors that contribute to heart disease.

### Limitations

This study has several limitations. The study cohort was primarily female and African American. Our analysis used posts from patients with at least 200 words in their Facebook posts, and therefore we cannot extrapolate about those who used social media less or did not consent to share; we used 200 words because prior work on using social media for predicting individuals’ traits determined that for good and stable predictive performance when working with social media data, data from users with 200 words or more on Facebook should be used [24,25]. Our sample was also limited to those with an ASCVD risk score in a single health system EMR, and therefore we may have missed individuals with a risk score in another EMR or that may not have had a risk score calculated in our EMR.

### Conclusion

We show that language from Facebook posts can be used to predict an individual’s 10-year risk for ASCVD. Specific information in posts could help to guide clinicians in better understanding lifestyles and behaviors, and in counseling patients about heart disease risk.

## Appendix

Multimedia Appendix 1 Twenty topics generated from our dataset.

## Abbreviations

ASCVD: atherosclerotic cardiovascular disease
AUC: area under the receiver operating characteristic curve
CV: cardiovascular
EMR: electronic medical record
LDA: latent Dirichlet allocation
LIWC: Linguistic Inquiry and Word Count
PCE: pooled cohort equations
