# Supplementary material for: Predicting Cardiovascular Risk Using Social Media Data: Performance Evaluation of Machine-Learning Models
Source: JMIR Cardio. 2021 Feb 19;5(1):e24473. doi: 10.2196/24473 (PMC8411430; doi:10.2196/24473)
Supplement: Multimedia Appendix 1 [file cardio_v5i1e24473_app1.docx]

| Topic number | Topic-associated words |
| --- | --- |
| 1 | babe, work, omg, lol, today, guy, home, can’t, wow, guys |
| 2 | I’m, feel, ppl, sick, smh, damn, :-(, hate, feeling, headache |
| 3 | people, life, don’t, make, cancer, hard, care, change, bad, real |
| 4 | car, snow, house, door, put, crazy, kids, man, wth, stop |
| 5 | Obama, black, president, mr, America, vote, history, law, country, Barack |
| 6 | Dinner, cheese, eat, made, food, breakfast, hot, drink, chicken, turkey |
| 7 | God, love, bless, amen, Lord, pray, heart, peace, faith, Jesus |
| 8 | lol, weekend, daughter, purple, nice, party, fun, cool, night, girls |
| 9 | Years, girl, year, days, baby, check, mom, ago, son, minutes |
| 10 | Happy, love, birthday, mom, baby, Christmas, son, year, sister, dad |
| 11 | Good, day, morning, Facebook, hope, safe, blessed, weather, enjoy, afternoon |
| 12 | Good, bed, sleep, night, home, ready, tired, long, early, tomorrow |
| 13 | School, high, Philadelphia, class, children, job, free, parents, support, church |
| 14 | Game, eagles, watch, play, win, team, tv, tonight, playing, movie |
| 15 | Alert, amber, child, active, updates, safe, November, police, sierra, found |
| 16 | Daily, affirmation, happening, November, philly, Saturday, Friday, October, Thursday, Sunday |
| 17 | Family, day, friends, gm, great, hope, tip, gn, things, success |
| 18 | Money, screwed, stupid, arrested, job, free, pay, company, society, business |
| 19 | Time, work, back, week, wait, tomorrow, days, weekend, Friday, hours |
| 20 | Phone, Facebook, read, post, call, page, friend, writing, status, write |
